# Modeling Faking in the Multidimensional Forced-Choice Format: The Faking Mixture Model

**DOI:** 10.1007/s11336-021-09818-6

**Published:** 2021-12-20

**Authors:** Susanne Frick

**Affiliations:** Department of Psychology, School of Social Sciences, Mannheim, Germany

**Keywords:** multidimensional forced-choice, faking, item response theory, mixture model

## Abstract

The multidimensional forced-choice (MFC) format has been proposed to reduce faking because items within blocks can be matched on desirability. However, the desirability of individual items might not transfer to the item blocks. The aim of this paper is to propose a mixture item response theory model for faking in the MFC format that allows to estimate the fakability of MFC blocks, termed the Faking Mixture model. Given current computing capabilities, within-subject data from both high- and low-stakes contexts are needed to estimate the model. A simulation showed good parameter recovery under various conditions. An empirical validation showed that matching was necessary but not sufficient to create an MFC questionnaire that can reduce faking. The Faking Mixture model can be used to reduce fakability during test construction.

**Supplementary Information:**

The online version supplementary material available at 10.1007/s11336-021-09818-6.

## Introduction

In many personality assessment contexts, individuals are motivated to obtain certain results. For example, when personality assessment is used in personnel selection, individuals may be motivated to distort their responses to make a favorable impression and get a job offer. In clinical assessment, individuals may be motivated to distort their responses to obtain a certain diagnosis or compensation. Such distortions are called *faking*. Although there is no agreed-upon definition of faking, the consensus is that it is a motivated behavior that occurs as an interaction between a person and a situation, and results in leaving an inaccurate or enhanced impression (MacCann et al., 2011). Thus, faking must be distinguished from unintentional response distortions such as self-deceptive enhancement (Paulhus, 2002), careless responding (Curran, 2016; Meade & Craig, 2012) or response styles (Paulhus, 1991; Wetzel et al., 2016). Most assessments are currently conducted with rating scale questionnaires. Faking seems to be quite prevalent on rating scales, resulting in increases of .1 to .6 *SD* in trait estimates (Birkeland et al., 2006; Viswesvaran & Ones, 1999) in real or simulated high-stakes situations.

### The Multidimensional Forced-Choice Format as a Remedy

The multidimensional forced-choice (MFC) format has been proposed as an alternative to rating scales in order to prevent faking. In the MFC format, several items measuring different attributes are combined into blocks. People have to rank-order the items within a block according to how well the items describe them. There are other variants, for example, selecting the items that describe oneself most and/or least (for an overview of different variants of the MFC format and how to model them, see Brown & Maydeu-Olivares, 2018b). In order to prevent faking in the MFC format, items with equal desirability are combined into blocks, such that all rank orders of items are equally desirable. In this case, items cannot be ranked by their desirabilities. This implies that estimates of item desirability are accurate at the group level and individuals will not differ in their evaluations of item desirability. By contrast, for rating scales, it is often theoretically obvious that higher (or lower) response options are indicative of desirable behaviors or traits.

Overall, using the MFC format has resulted in less evidence of faking than when rating scales are used (Cao & Drasgow, 2019). This has been particularly apparent when faking was evaluated on the group level via mean differences between groups that were instructed to respond honestly compared with groups that were instructed to fake (e.g., Christiansen et al., 2005; Jackson et al., 2000). So far, only two studies have examined faking on the individual level by correlating rank orders of individuals between honest and faking conditions (Heggestad et al., 2006; Wetzel et al., 2021). Heggestad et al. (2006) found similar faking for the MFC format and rating scales. Wetzel et al. (2021) found that rank orders changed when faking, for both the MFC format and the rating scales. The finding that MFC questionnaires are still fakable to some extent raises the question of whether item matching can be improved.

### Matching and Item Interactions

Indeed, there is evidence that poorly matched MFC blocks elicit higher agreement as to the optimal rank order than closely matched blocks (Hughes et al., 2021). Further, item desirability might differ across contexts. For example, in an instructed faking study, participants reported higher agreeableness when applying for a job as a nurse compared to as a manager (Pauls & Crost, 2005). Thus, if desirability values were obtained with a manager sample, items that are positively keyed toward agreeableness will increase in desirability and items that are negatively keyed will decrease in desirability when evaluated by nurse applicants. A questionnaire that is fake-proof for one sample or assessment context might not necessarily be so for another one.

Current procedures for matching items to MFC blocks are based on the assumption that item desirability is roughly the same when items are answered separately (i.e., in single-stimulus formats) or relative to each other (i.e., in an MFC format). However, item desirability might change or be evaluated in a more differentiated manner within the context of item blocks (Feldman & Corah, 1960; Hofstee, 1970). In line with this idea, some researchers have argued that desirability should be viewed as a property of response options rather than of items (Kuncel & Tellegen, 2009). More generally, several researchers have observed changes in item parameters or slight changes in constructs between single-stimulus and MFC formats (e.g., Guenole et al., 2018; Wetzel & Frick, 2020) and even changes in item parameters within the MFC format, depending on which items were combined into blocks (Lin & Brown, 2017). To improve the construction of fake-proof MFC questionnaires, a method is needed to estimate the *fakability* of each MFC block (i.e., the extent to which it can be faked). However, current methods only allow the fakability of the whole questionnaire to be assessed (i.e., for all blocks simultaneously). Moreover, they make assumptions that might be wrong.

### Assessing Fakability

The fakability of a questionnaire is most often evaluated by comparing responses and trait estimates from a real or simulated high-stakes situation with those from a low-stakes situation. Usually, traits in a high-stakes situation are estimated by fixing the item parameters to the estimates obtained in a low-stakes situation (e.g., Wetzel et al., 2021). This approach, termed the *trait shift approach* in the following, can be applied to both rating scales and MFC questionnaires. It shows how estimated traits would change in practice, when the true model was naively applied. From a modeling perspective, the trait shift approach assumes a shift in the response process, similar to many models formulated for response styles (Henninger & Meiser, 2020; Plieninger & Heck, 2018). As a shift model, the trait shift approach assumes that both the content trait and faking influence all responses. Specifically, the shift is weighted by the item loading, and, across all items measuring the same trait, fakers are assumed to increase their trait level to the same extent. These assumptions might be wrong.

Building on the trait shift approach, Pavlov et al. (2019) proposed that faking scores should be regressed on honest scores to model both a tendency to fake and moderation effects on it. One drawback of their approach is that it relies on observed scores, which are inherently less reliable for MFC than for rating scales (Brown & Maydeu-Olivares, 2018a; Frick et al., in press). Further, none of the current modeling approaches for faking in MFC questionnaires allows the fakability of individual items or blocks to vary. Thus, they allow faking to vary by person, but fakability is the same for all items or blocks. From a basic research perspective, this assumption might be wrong. From a practical perspective, these approaches allow the fakability of the questionnaire to be examined as a whole, but they do not show how to modify the questionnaire to reduce its fakability.

Within item response theory, Böckenholt (2014) proposed the retrieve–edit–select framework in which responses based on initial retrieval are edited in a certain direction. Leng et al. (2019) proposed the retrieve–deceive–transfer model, which incorporates both the retrieval of socially desirable information and the editing of responses. However, these models are formulated for single-stimulus items, for which a desirable direction can clearly be identified. For MFC blocks, a desirable rank-order cannot easily be identified a priori.

To improve the construction of fake-proof MFC questionnaires, an item response model for faking tailored to the MFC format is needed. Such a model would allow researchers to evaluate fakability on the block level and discard or modify blocks accordingly. More generally, it would show which properties describe less fakable blocks. By focusing on the block level, it would reflect the MFC response process better than the current approaches.

### Aim

The aim of this paper is to propose a model for faking in the MFC format that allows the fakability of individual MFC blocks to be evaluated, considering that the tendency to fake varies between individuals. Hence, the main purpose of the model is to help construct more fake-proof questionnaires.

### Effects of Faking on MFC Rank Orders

In a high-stakes situation, respondents do not necessarily fake all items or all traits (Ziegler, 2011). Estimates of how many people fake their answers range from 14 to 40%, with the consensus being that around 25% fake (MacCann et al., 2011). Therefore, it is appropriate to conceptualize the response process in a high-stakes situation as a mixture of two processes: an honest responding process and a faking process. Both processes have implications for the rank orders selected on MFC blocks.

In a high-stakes situation, respondents might select the same rank order as in a low-stakes situation for several reasons: (a) they are not motivated to fake, (b) they do not need to fake, because their responses are already desirable, or (c) the MFC block is closely matched (i.e., all items are equally desirable). When facing a closely matched block, respondents might react in several ways: First, respondents might decide to give an honest response (Berkshire, 1958), or second, they might perceive their honest response as more desirable (Gordon, 1951).

Alternatively, in a high-stakes situation, respondents might select rank orders that do not reflect their content trait levels but might instead reflect what they perceive as desirable. In this case, the distribution of rank orders can show how well the items were matched. For a closely matched block, respondents who are motivated to fake might either respond randomly or retrieve more information about the desirabilities of the items. For example, they might evaluate the item desirabilities in a more differentiated manner (Feldman & Corah, 1960; Hofstee, 1970) in order to be able to rank the items on the basis of their desirabilities. To the observer, both choices result in a uniform distribution of rank orders across respondents. In line with this idea, Kuncel and Borneman (2007) used rating scale items that showed bivariate or trivariate response distributions under faking instructions as indicators of faking.

By contrast, respondents facing a poorly matched block will agree on which rank orders are preferable. To the observer, the distribution of rank orders will be skewed. For example, there might be one rank order that is clearly most desirable and therefore has the highest frequency. Or, there might be one item in a block that is most (or least) desirable, leading to higher frequencies of rank orders that favor (or disfavor) this item. Indeed, in one study, agreement about which rank order should be preferred was higher the worse the matching was (Hughes et al., 2021).

## The Faking Mixture model

The proposed model for analyzing faking in the MFC format is a mixture model that allows the occurrence of faking to vary by block and by person. Therefore, I will call it the *Faking Mixture model* in the following. The Faking Mixture model can be used to estimate the fakability of blocks, the probabilities of different rank orders when faking and individuals’ faking tendencies. Given current computing capabilities and programs, within-subject data from both a low- and a high-stakes situation are needed for model estimation. The current purpose of the model is thus to improve MFC test construction, but not to correct content trait estimates for faking.

### Model Formulation

The proposed model is a mixture model: In a high-stakes situation, individuals base their responses *either* on the desirability *or* on the content trait. By distinguishing between two response processes, it makes explicit that not all individuals fake all blocks when in a high-stakes situation. This is in contrast to a shift model, in which both the content trait and faking influence all responses. For simplicity, in the following, I will use the terms *honest* responding for responses that are based on the content trait and *faking* for responses that are based on desirability. However, the assessment situation and the sample both shape, for example, whether honest responding is influenced by socially desirable responding. The model properties are defined by the following model equations.

The Faking Mixture model models the probability of selecting a certain rank order. This is in contrast to most IRT models for MFC data that can be expressed in terms of pairwise preferences (Brown, 2016a), for an exception, see Joo et al. (2018). Let *k* index the item block within the questionnaire and $r=1 \ldots R$ the possible rank orders (i.e., the permutations). For a block of size *B*, there are $R=B!$ possible rank orders (permutations). For example, if $B=3$ the possible rank orders are 1–2–3, 1–3–2, 2–1–3, 2–3–1, 3–1–2, and 3–2–1. Further, let *F* be an indicator variable that takes on a value of 1 if a person fakes and 0 otherwise. Then, the probability that the observed rank order *X* for person *j* on block *k* takes on a value of *r* can be described as follows:

1 $$\begin{aligned} P(X_{jk}=r) = P(F_{jk}=1)P(X_k=r|F_{jk}=1) + P(F_{jk}=0)P(X_{jk}=r|F_{jk}=0) \end{aligned}$$

The probability of selecting rank order *r* for block *k* when faking, termed the *rank-order probability* $P(X_k=r|F_{jk}=1)$, varies only by block but not by person. To reflect this, the person subscript *j* is dropped. The rank-order probabilities are linked to continuous, unconstrained rank-order parameters $\beta _{kr}$:

2 $$\begin{aligned} P(X_k=r|F_{jk}=1) = \frac{\exp (\beta _{kr})}{\sum _{u=1}^{R}\exp (\beta _{ku})} \end{aligned}$$

For identification, and without loss of generality, the parameter for the first rank order in each block is fixed to zero: $\beta _{k1}\equiv 0$. As a consequence of this indeterminacy, the rank-order parameters $\beta _{kr}$ cannot be interpreted in absolute terms but only relative to each other. For example, a relatively high rank-order parameter $\beta _{kr}$ translates into a high probability of selecting this rank order when faking. It is convenient to focus on the rank-order probabilities, because they can be interpreted in absolute terms. If the block is closely matched (i.e., the differences between the item desirabilities are small), the rank-order probabilities will be approximately equal. If matching is poor, the rank-order probabilities will be relatively higher for one or several rank orders. The $R=B!$ rank-order parameters $\beta _{kr}$ are not linked to the *B* items; hence, they cannot be expressed in terms of individual item desirabilities. However, in this way, the rank-order probabilities reflect differences in item desirabilities within a block and hence capture item interactions and the relative nature of the MFC responses.

The probability of faking a block depends on the block fakability $\alpha _k$ and a faking trait $\theta _j$ modeled via a probit link:

3 $$\begin{aligned} P(F_{jk}=1) = \Phi \left( \theta _j + \alpha _k\right) \end{aligned}$$

The block fakability $\alpha _k$ is not an independent parameter but is obtained from the rank-order parameters $\beta _{kr}$. It is the quantile of the standard normal distribution at the sum of squares of the rank-order probabilities. The quantile is used to transform the sum of squares from the probability scale into a continuous scale. If $\Phi (x)$ denotes the cumulative standard normal distribution function, evaluated at *x*, and $\Phi ^{-1}$ its inverse, then:

4 $$\begin{aligned} \alpha _k = \Phi ^{-1}\left( \sum _{r=1}^{R}\left( P(X_{k}=r|F_{jk}=1) - {M}\left[ \varvec{P(X_{k}|F_{jk}=1)}\right] \right) ^2\right) \end{aligned}$$

Thus, the block fakability $\alpha _k$, and with it the probability of faking a block, increases with greater variance in the rank-order probabilities. If a preferable ranking can be clearly identified for a block, the block fakability $\alpha _k$ is higher, and respondents are more likely to fake this block. If items within a block cannot be ranked by desirability, the block fakability $\alpha _k$ is lower, and respondents are more likely to respond honestly (i.e., based on the content traits).

The faking trait $\theta _j$ captures the propensity to fake, both in terms of the sample mean and interindividual variation around it. Even for closely matched blocks, a high faking trait leads to high faking probabilities. This can capture the fact that a situation might strongly motivate respondents to fake, even when they differ in which rank orders they perceive as desirable.

The response probabilities when responding honestly $P(X_{jk}=r|F_{jk}=0)$ follow an IRT model for MFC data. For my applications, I chose the Thurstonian IRT model (Brown & Maydeu-Olivares, 2011), because it is the most broadly applicable model and is based on a linear factor structure, which is commonly assumed for personality questionnaires. In the Thurstonian IRT model, it is assumed that a latent, continuous value called *utility* underlies the responses. For personality questionnaires, the utility reflects how useful the item is for describing the person. The utility *t* of person *j* on item *i* is a linear function of a latent content trait $\eta _j$, weighted with an item loading $\lambda _i$ and having an intercept $\mu _i$ and an error term $\varepsilon _{ji}$:

5 $$\begin{aligned} t_{ji} = \mu _i + \lambda _i\eta _j + \varepsilon _{ji} \end{aligned}$$

The errors of item *i* are normally distributed with $\varvec{\varepsilon _{i}} \sim N(0,\psi _i)$.

According to Thurstone’s law of comparative judgment (Thurstone, 1927), items within blocks are ranked according to the magnitude of their utilities. Response probabilities for rank orders can be calculated by following the formulation by Yousfi (2018):

6 $$\begin{aligned} P(X_{jk}=r|F_{jk}=0) = \int _{0}^{\infty }\int _{0}^{\infty }\ldots \int _{0}^{\infty }{MVN}\left( \varvec{A t_{jk}}(r), \varvec{A\psi _k^2}(r)\right) d\varvec{A t_{jk}}(r) \end{aligned}$$

Vectors of utilities $\varvec{t_{jk}}$ and of error variances $\varvec{\psi _k^2}$ are sorted in descending order, according to the selected rank order *r*. Then, the response probability is the area under the multivariate density where the first utility is larger than the second, the second is larger than the third and so forth. This order is ensured by the limits of the integral and the comparison matrix *A*. For example, for a block of size $B=3$:

7 $$\begin{aligned} {\varvec{A}}_{B=3} = \begin{pmatrix} 1 \quad &{} -1 \quad &{} 0 \\ 0 \quad &{} 1 \quad &{} -1 \end{pmatrix} \end{aligned}$$

Within a structural equation framework, Thurstonian IRT models can easily be estimated via limited information methods (Brown & Maydeu-Olivares, 2011; Maydeu-Olivares, 1999; Maydeu-Olivares & Brown, 2010). However, estimating item parameters on the basis of response probabilities for rank orders is hardly feasible with the current computing capabilities and programs that are available to the usual researcher. The multiple integral in Eq. (6) can be solved by numerical integration (Genz & Bretz, 2009), which is so far implemented only in R, MATLAB, and Fortran. However, this numerical integration is apparently too complex to be used for item parameter estimation as implemented in mirt (Chalmers, 2012), resulting in unreasonable computing times and estimates. Bayesian estimation programs, such as JAGS (Plummer, 2017) or Stan (Carpenter et al., 2017), do not include such a function.

As a solution, response probabilities when responding honestly are estimated with data that can be assumed to primarily reflect the content traits. For this, item and trait parameters are estimated in a structural equation framework in a first step, and the response probabilities are calculated with these parameters. The Faking Mixture model is then estimated in a second step, by using data from the same respondents in a high-stakes situation, with the response probabilities that occur when the respondents answer honestly $P(X_{jk}=r|F_{jk}=0)$ fixed to those obtained in the first step.

Filling in the mixture Eq.  (1) with the above specifications gives the response probability under the Faking Mixture model, where the $P(X_{jk}=r|F_{jk}=0)$ are calculated a priori:

8 $$\begin{aligned} P(X_{jk}=r | \theta _j) = \Phi \left( \theta _j + \alpha _k\right) \frac{\exp (\beta _{kr})}{\sum _{u=1}^{R}\exp (\beta _{ku})} + \left( 1 - \Phi \left( \theta _j + \alpha _k\right) \right) P(X_{jk}=r|F_{jk}=0) \end{aligned}$$

### Implementation

The Faking Mixture model is implemented in a Bayesian framework with the following priors and hyperpriors:

9 $$\begin{aligned} \varvec{\beta }&\sim N\left( 0,SD(\varvec{\beta }) \right) \nonumber \\ \varvec{\theta }&\sim N \left( M(\varvec{\theta }), SD(\varvec{\theta }) \right) \nonumber \\ M(\varvec{\theta })&\sim N(1,2) \nonumber \\ Var(\varvec{\theta })&\sim Inverse\text { }Gamma(1.5, 1) \nonumber \\ Var(\varvec{\beta })&\sim truncated\text { }N(5,10,0,15) \end{aligned}$$

Thus, these hyperpriors allow the mean block fakability (determined by Var($\varvec{\beta }$)) and the mean and the variance of the faking trait to be estimated from the data, thereby reducing prior sensitivity (Fox, 2010). In preliminary simulations, the priors and hyperpriors were fine-tuned to ensure model convergence and good recovery under various conditions. The parameters can be sampled from the posterior (e.g., via Hamiltonian Monte Carlo sampling as implemented in Stan; Stan Development Team, 2020b). The Stan Code for estimating the Faking Mixture model can be found at https://osf.io/wfhz4/.

## Simulation Study: Parameter Recovery

A small simulation study was conducted to evaluate parameter recovery for the Faking Mixture model. Response probabilities when responding honestly ($P(X_{jk}=r|F_{jk}=0)$) were based on the true item and trait parameters. One thousand replications were conducted. The R code used to run and analyze the simulation, along with the simulation materials and results, can be found at https://osf.io/wfhz4/.

### Simulation Design

Responses were simulated for 500 respondents, 20 blocks of three items each, and five traits. The five traits were drawn from a multivariate normal distribution with a mean vector of 0, variances of 1, and correlations set to meta-analytic estimates for the Big Five (neuroticism, extraversion, openness, agreeableness, and conscientiousness) as reported by van der Linden et al. (2010), see Table 1. The faking trait $\varvec{\theta }$ was drawn from a normal distribution, independent of the content traits. Note that although this might be unrealistic, it represents a correctly specified model as long as the faking trait and the content traits cannot be estimated at the same time.

**Table 1:** Correlations used in the simulation study

Trait	E	O	A	C
N	$-$.36	$-$.17	$-$.36	$-$.43
E		.43	.26	.29
O			.21	.20
A				.43

*Note.* N = neuroticism, E = extraversion, O = openness, A = agreeableness, C = conscientiousness. These are meta-analytic correlations between the Big Five as reported by van der Linden et al. (2010)

Three factors were varied and completely crossed, that is, all possible combinations of the factor levels were realized: fakability, faking trait mean $M(\varvec{\theta })$, and faking trait variance $Var(\varvec{\theta })$. The *fakability* factor was varied with the levels of high and low. For high and low fakability, the rank-order parameters $\beta _{kr}$ were drawn from $U(-4,4)$ and $U(-2,2)$, respectively. For low fakability, 90% of the highest rank-order probabilities per block are between .3 and .6 ($M=.4$, $SD=.1$). For high fakability, 90% are between .4 and .9 ($M=.6$, $SD=.2$). The *faking trait mean* factor was varied with the levels 0, 1, and 2. With these levels, across blocks, the mean probability of faking ranges from .15 for low fakability and $M(\varvec{\theta })=0$, mimicking the lower estimate of the propensity to fake from MacCann et al. (2011), to .90 for high fakability and $M(\varvec{\theta })=2$, representing the extreme end of faking. The *faking trait variance* factor was varied with the levels 0.2, 0.5, and 1. In relation to the content trait variance of 1, 0.2 is a typical variance for a trait that captures response biases (Billiet & McClendon, 2000; Plieninger & Heck, 2018).

### Data Generation

To simulate the data for honest responding, item parameters were drawn from the following distributions: $\varvec{\mu } \sim U(-1,1)$, $\varvec{\lambda } \sim U(.65, .95)$. These are typical values for standardized item utilities with good measurement properties (Brown & Maydeu-Olivares, 2011). To ensure that the loadings allow for the recovery of normative trait levels, they were redrawn until there were no linear dependencies between loadings within a block. If there are linear dependencies within a block, for example, because all loadings have approximately the same size or are multiples of each other, the Thurstonian IRT model is not identified (see Brown, 2016a). In addition, the direction of factor loadings was set such that half of the pairwise item comparisons within blocks were between differently keyed items (i.e., one negative, one positive factor loading). This has been shown to aid the recovery of normative trait levels in simulation studies (e.g., Brown & Maydeu-Olivares, 2011; Bürkner et al., 2019; Frick et al., in press). To simulate standardized utilities, the error variances were set to $\psi _i^2 = 1 - \lambda _i^2$. Errors were drawn from $\varvec{\varepsilon _{i}} \sim N(0,\psi _i)$. Using Eq.  (5), utilities were calculated given the traits, item parameters and errors. To obtain the responses when responding honestly, the rank order of utilities within each block was determined for each person.

To simulate the data in a high-stakes situation, the parameters for the Faking Mixture model were drawn according to the respective condition of the simulation design. Next, the probabilities of faking the blocks were calculated using Eq.  (3), for each person and each block. To determine whether a block was faked by a person, a dichotomous outcome was drawn from a binomial distribution with the respective probability. To obtain the response for a faked block, a categorical outcome was drawn from a multinomial distribution with the rank-order probabilities $P(X_k=r|F_{jk}=1)$ for this block. To obtain the responses in a high-stakes situation, for each faked block, the response when responding honestly was replaced by the respective categorical outcome.

Response probabilities when responding honestly were calculated with Eq. (6) with the true item and trait parameters. Then, the Faking Mixture model was fit to the faking data, given the response probabilities when responding honestly calculated in the previous step. R (R Core Team, 2020) was used for data generation and analysis along with the packages rstan (Stan Development Team, 2020a), MASS (Venables & Ripley, 2002), psych (Revelle, 2019), and mvtnorm (Genz & Bretz, 2009; Genz et al., 2020). Stan (Carpenter et al., 2017) was used for model estimation. Three chains were run with 3500 iterations out of which the first 750 were discarded. In preliminary simulations, these values were selected to ensure convergence and sufficient parameter recovery.

### Data Analysis

The coverage of 95% posterior intervals was computed along with the correlation between true and estimated values, mean bias and variance in bias for posterior medians of (a) the main parameters: rank-order parameters $\beta _{kr}$ and the faking trait $\varvec{\theta }$, (b) the hyperparameters: faking trait mean $M(\varvec{\theta })$, faking trait variance $Var(\varvec{\theta })$, and rank-order parameter variance $Var(\varvec{\beta })$, and (c) the derived parameters: block fakabilities $\alpha _k$, and rank-order probabilities $P(X_k=r|F_{jk}=1)$. Within each condition, I evaluated whether the absolute levels of parameter recovery were satisfactory. Furthermore, for each dependent variable, I calculated the explained variance within an ANOVA framework to see whether recovery varied by the manipulated simulation factors. In contrast to the *F*-test, explained variance is descriptive and insensitive to heterogeneous variances, which occurred in some of the simulation conditions.

### Results and Discussion

For all parameters, the coverage of the 95% posterior intervals was almost perfect across conditions ($\ge .95$) and did not vary systematically with the manipulated factors (Table 2). Mean bias was generally low in relation to the scale of the respective parameter (Table 3). The hyperparameters, faking trait mean $M(\varvec{\theta })$ and variance $Var(\varvec{\theta })$, and the variance of the rank-order parameters $Var(\varvec{\beta })$ were recovered well across the simulated conditions.

**Table 2:** Variance explained in % by the manipulated factors in the simulation study

Factor	Main parameters							
	$\theta _{fj}$				$\beta _{kr}$			
	*r*	MB	SDB	95%	*r*	MB	SDB	95%
$Var(\varvec{\beta })$	1	0	1	0	0	31	63	0
$Mean(\varvec{\theta })$	18	8	14	0	63	4	24	6
$Var(\varvec{\theta })$	67	1	72	3	2	0	0	0
$Var(\varvec{\beta })\times Mean(\varvec{\theta })$	8	0	6	0	7	0	0	1
$Var(\varvec{\beta })\times Var(\varvec{\theta })$	0	0	0	0	1	0	0	0
$Mean(\varvec{\theta })\times Var(\varvec{\theta })$	1	5	2	0	3	0	1	0
$Var(\varvec{\beta })\times Mean(\varvec{\theta })\times Var(\varvec{\theta })$	0	0	1	0	1	0	0	0
Residuals	6	85	5	96	23	65	13	93

	Hyperparameters							
	$Mean(\varvec{\theta })$		$Var(\varvec{\theta })$		$Var(\varvec{\beta })$			
	B	95%	B	95%	B	95%		
$Var(\varvec{\beta })$	0	0	0	0	1	0		
$Mean(\varvec{\theta })$	0	0	0	0	4	1		
$Var(\varvec{\theta })$	0	1	1	1	0	0		
$Var(\varvec{\beta })\times Mean(\varvec{\theta })$	0	0	0	0	1	0		
$Var(\varvec{\beta })\times Var(\varvec{\theta })$	0	0	0	0	0	0		
$Mean(\varvec{\theta })\times Var(\varvec{\theta })$	0	0	0	0	0	0		
$Var(\varvec{\beta })\times Mean(\varvec{\theta })\times Var(\varvec{\theta })$	0	0	0	0	0	0		
Residuals	99	99	99	99	94	99		

	Derived parameters							
	$\alpha _k$				$P(X_k=r|F_{jk}=1)$			
	*r*	MB	SDB	95%	*r*	MB	SDB	95%
$Var(\varvec{\beta })$	30	0	19	0	27	5	18	1
$Mean(\varvec{\theta })$	18	0	37	0	32	76	62	1
$Var(\varvec{\theta })$	0	0	0	0	2	2	2	0
$Var(\varvec{\beta })\times Mean(\varvec{\theta })$	10	0	6	0	18	7	6	0
$Var(\varvec{\beta })\times Var(\varvec{\theta })$	0	0	0	0	1	1	0	0
$Mean(\varvec{\theta })\times Var(\varvec{\theta })$	0	0	1	0	3	4	3	0
$Var(\varvec{\beta })\times Mean(\varvec{\theta })\times Var(\varvec{\theta })$	0	0	0	0	2	1	0	0
Residuals	42	99	38	100	16	4	9	98

*Note.* *r* = correlation, MB = Mean bias, SDB = *SD* bias, 95% = coverage of 95% posterior intervals, $Var(\beta )$ = fakability

**Table 3:** Mean bias across conditions in the simulation study

Variable	Mean	*SD*	lower	upper
$\theta _{j}$	0.00	0.05	$-$0.09	0.08
$\beta _{kr}$	$-$0.24	0.20	$-$0.63	0.01
$Mean(\varvec{\theta })$	0.00	0.05	$-$0.08	0.09
$Var(\varvec{\theta })$	0.01	0.07	$-$0.10	0.12
$Var(\varvec{\beta })$	0.05	1.02	$-$1.66	1.84
$\alpha _k$	0.00	0.03	$-$0.05	0.04
$P(X_k=r|F_{jk}=1)$	0.00	0.00	0.00	0.00

*Note.* lower = 5% quantile, upper = 95% quantile, $Var(\beta )$ = fakability

**Fig. 1:**
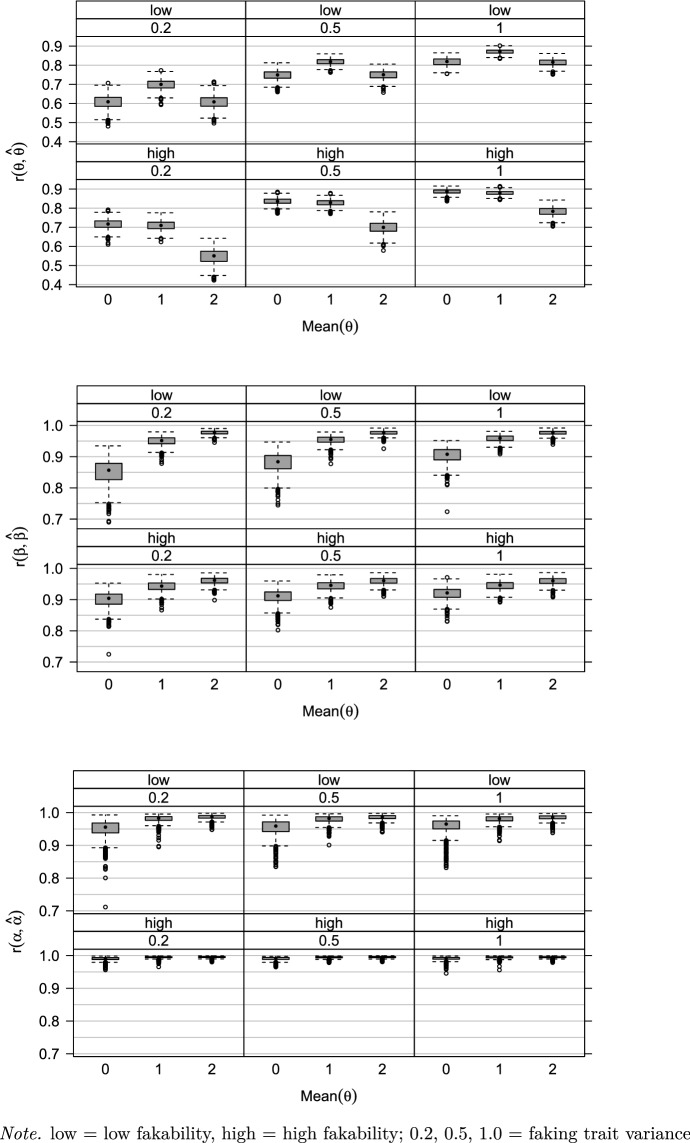
Correlations between true and estimated parameters in the simulation study by condition

Regarding the main parameters, the manipulated factors showed some effects (Table 2). For the faking trait $\varvec{\theta }$, as would be expected for any latent trait, the faking trait variance factor $Var(\varvec{\theta })$ explained the largest amount of variance in the correlations (67%, Table 2) and *SD*s of the bias (72%): The correlations (see Fig. 1) and *SD*s of the bias increased (with means of 0.34, 0.44, and 0.54) as the faking trait variance $Var(\varvec{\theta })$ increased. Moreover, 18% of the variance in the correlations and 14% in the *SD*s of the bias were explained by the faking trait mean factor $M(\varvec{\theta })$ (Table 2): The correlations (Fig. 1) were highest and the *SD*s of the bias were lowest for a faking trait mean $M(\varvec{\theta })$ of 1, followed by means of 0 and 2 (the mean *SD*s of the bias were: 0.40, 0.43, and 0.48, for $M(\varvec{\theta })=$ 1, 0, and 2, respectively). Such values are to be expected because differences in $\varvec{\theta }$ are harder to detect the more extreme the mean is, because, for a high (low) faking trait mean, most (almost no) individuals are predicted to fake.

For the rank-order parameters $\beta _{kr}$, the faking trait mean factor $M(\varvec{\theta })$ explained the largest amount of variance in the correlations (63%, Table 2) and the second largest amount of variance in the *SD*s of the bias (24%). The correlations (Fig. 1) increased and the *SD*s of the bias decreased (with means of 1.07, 0.78, and 0.63) with higher faking trait means $M(\varvec{\theta })$. Thus, the rank-order parameters are better recovered the more the sample is inclined to fake. There was a systematic negative bias for the rank-order parameters $\beta _{kr}$: 94% of the mean biases were $<0$. This negative bias emerged by design because the only effect of the $\beta _{kr}$ is through their transformation into rank-order probabilities $P(X_k=r|F_{jk}=1)$, which add up to 1. Consequently, there is some dependency even among $k-1$ free rank-order parameters $\beta _{kr}$. If any of the $\beta _{kr}$ within a block have a positive bias, all others must have a negative bias, leading to a negative mean bias. Bias in absolute terms increased with fakability (with means of -0.13 for low and -0.36 for high fakability), explaining the largest amount of variance (31%, Table 2), because fakability was operationalized as variance in the rank-order parameters. Similarly, the *SD*s of the bias increased with higher fakability (with means of 0.54 and 1.12 for low and high fakability, respectively), explaining 63% of the variance (Table 2). Still, in absolute terms, the bias across conditions was small (Table 3) and the correlations between the true and estimated rank-order parameters were almost perfect (mean of .94, $SD=.04$, Fig. 1). The effects for the block fakability parameters $\alpha _k$ mirrored those found for the rank-order parameters $\beta _{kr}$. For the rank-order probabilities, differences between the conditions were negligible in size. Additional information on parameter recovery can be found in Figures S1–S16.

To sum up, the simulation showed that the parameters of the Faking Mixture model could be recovered well across the simulated conditions. As to be expected, the reliability of the faking trait increased as the variance increased. There was a slight dependency between the recovery of the faking trait and of the rank-order parameters, such that recovery for the latter was better with a higher faking trait mean and vice versa. However, this dependency was small enough to be negligible.

## Empirical Validation

To validate the Faking Mixture model and to illustrate its application, I used a dataset that was already analyzed by Wetzel et al. (2021) with the trait shift approach. Participants in this sample filled out an MFC personality questionnaire under both instructions to be honest and instructions to fake good. They were randomly assigned to either a version of the questionnaire in which all items within blocks were matched for desirability or a version in which some blocks were not matched (termed mixed blocks in the following). This dataset allowed the Faking Mixture model to be validated: If the Faking Mixture model works, mixed blocks should have higher fakability parameters than matched blocks. Further, rank-order probabilities for mixed blocks should favor rank orders in which the most desirable item is ranked highest.

The R code and data used in this empirical validation can be found at https://osf.io/wfhz4/.

### Method

#### Sample and Procedure

The sample consisted of two subsamples: one laboratory sample and one sample from an online access panel. In both subsamples, participants were remunerated for their participation and some participants were excluded due to data quality checks (for details, see Wetzel & Frick, 2020). The final sample consisted of 1244 participants. There were $N=592$ participants in the group with the matched version of the questionnaire, called MFC-matched (*M*(age) = 23.40, *SD*(age) = 4.10, 63% female, 0.3% transgender) and $N=652$ participants in the group with the partly mixed version of the questionnaire, MFC-mixed (*M*(age) = 23.39, *SD*(age) = 4.34, 65% female, 0.2% transgender).

The procedure was identical in the two groups. Participants filled out an MFC personality questionnaire first under instructions to be honest. Then, they filled out other personality questionnaires and questions about external criteria. Afterward, they received instructions to fake good and were asked to fill out the following questionnaire in accordance with the instructions. Then, they filled out the MFC personality questionnaire again. The fake good instructions asked them to imagine they were interested in a place in the Master’s program of psychology at a German university and that the following personality questionnaire was part of the application procedure. The instructions detailed which attributes the university was looking for in their students, which amounted to low levels of neuroticism and high levels on the other Big Five Traits (i.e., extraversion, openness, agreeableness and conscientiousness). For a detailed description of the faking instructions, see Wetzel et al. (2021); for more information about the sample and other measures, see Wetzel and Frick (2020).

#### Measures

The Big Five Triplets (BFT; Wetzel & Frick, 2020), an MFC questionnaire measuring the Big Five traits, were used. The BFT are available in German and English from https://osf.io/ft9ud/. The BFT consist of 20 blocks of three items each. During test construction, the social desirability of the individual items in a larger item pool was rated by 33 psychology students on a 5-point rating scale. Social desirability was defined as fulfilling societal norms and expectations, accompanied by three examples. The blocks of the BFT were matched by these desirability ratings. For example, the first triplet contains items that were all rated as socially undesirable. The BFT contain three socially undesirable, four neutral, and 13 socially desirable blocks. For the MFC-matched version, the original version of the BFT was used. Hence, the term *matched* in MFC-matched means that the items were matched by their desirability ratings. Whether those blocks are matched such that their true desirabilities are equal is an empirical question examined in the current study. To obtain the questionnaire version used for MFC-mixed, for each of the seven socially undesirable and neutral triplets, one item was replaced by a socially desirable item from the larger item pool that was not part of the original MFC-matched version. Thus, the two versions differed only in these seven items.

#### Data Analysis

First, the Thurstonian IRT model was fit to the data under the instructions to respond honestly, separately for MFC-mixed and MFC-matched, using Mplus. In contrast to Brown and Maydeu-Olivares (2011), the models were fit with a restriction on the intercepts to make the response probabilities on the block level add up to one. Second, the response probabilities were calculated with Eq.  (6). Third, the Faking Mixture model was fit to the faking data from MFC-mixed and MFC-matched. I will use the Faking Mixture model that was fit to the data from MFC-matched to illustrate how the model parameters can be interpreted.

To test whether fakability was higher for blocks with mixed desirability, I fit the Faking Mixture model to data from both MFC-matched and MFC-mixed. In this model, the rank-order parameters $\beta _{kr}$ were set equal for blocks that contained the same items in the two versions (Blocks 8–20), and they were estimated separately for blocks that differed between the two versions (Blocks 1–7). The rank-order parameters for blocks containing different items should not be set equal, but they can still lead to approximately equal block fakability parameters $\alpha _k$. To test for differences in the block fakability parameters $\alpha _k$ (for Blocks 1–7), these differences were included in the model so that 95% posterior intervals could be obtained for them. This is preferable to testing for differences between parameter estimates outside the model. Next, I examined whether rank orders favoring the desirable item in MFC-mixed had higher rank-order probabilities than in MFC-matched.

### Results

#### Convergence

The Thurstonian IRT models for MFC-matched and MFC-mixed showed excellent fits according to the RMSEA, with RMSEA values of .036 and .038, respectively, and acceptable fits according to the SRMR, with SRMR values of .089 and .093. Therefore, response probabilities when responding honestly could be calculated with these models. Due to estimation errors in the Mplus parameters, about half of all response probabilities did not add up to one in the seventh decimal place. Therefore, all response probabilities were rescaled by dividing them by the sum of the probabilities across the rank orders.

For the Faking Mixture models, to achieve convergence in both MFC-matched and MFC-mixed, I fixed the faking trait variance $Var(\varvec{\theta })$ to 0.25, which is a typical variance for a trait capturing response biases (Billiet & McClendon, 2000; Plieninger & Heck, 2018), and the variance of the rank-order parameters $Var(\varvec{\beta })$ to 4, which allows both low and high fakability parameters for individual blocks. Thus, I used a hyperprior only for the faking trait mean $M(\varvec{\theta })$. Six chains with 5000 iterations were run, out of which the first 2500 were discarded. I checked convergence via convergence criteria obtained from rstan, namely $\hat{R} < 1.01$ and the effective sample size divided by the true sample size larger than .001. I also visually inspected plots of posterior densities, of the autocorrelation and of the running mean across iterations. If not otherwise stated, I report posterior medians and 95% posterior intervals.

#### Descriptive Results

The faking trait $\varvec{\theta }$ was distributed with a mean of 2.97 [2.82; 3.14] and a variance of 0.29 [0.26; 0.33]. To better grasp the extent of faking, one can calculate the percentage of faking probabilities $\Phi \left( \theta _j + \alpha _k\right)$ that were $>.5$ for each block. This shows how many participants are predicted to fake. The high faking trait mean along with the percentages of participants predicted to fake (Table 4) show that the faking instructions were effective in motivating participants to fake. Due to the low variance in the faking trait, estimates of $\theta _j$ are not very reliable (see also the simulation study). Therefore, they should not be used to investigate the validity of the faking trait via correlations with other traits or criteria.

**Table 4:** Block fakabilities $\alpha _k$ and percentage of participants predicted to fake in MFC-matched

Block	Median	$2.5\%$	$97.5\%$	Predicted
3	-2.01	-2.19	-1.86	99
12	-1.95	-2.11	-1.79	99
1	-1.87	-2.02	-1.74	99
17	-1.86	-2.01	-1.72	99
10	-1.80	-1.95	-1.66	99
16	-1.77	-1.95	-1.62	100
9	-1.70	-1.83	-1.58	100
8	-1.68	-1.81	-1.56	100
11	-1.67	-1.80	-1.54	100
20	-1.61	-1.74	-1.50	100
13	-1.50	-1.65	-1.37	100
15	-1.41	-1.55	-1.29	100
5	-1.26	-1.38	-1.14	100
2	-1.25	-1.38	-1.14	100
14	-1.25	-1.37	-1.13	100
6	-1.17	-1.28	-1.06	100
18	-1.11	-1.23	-1.00	100
7	-1.09	-1.21	-0.97	100
19	-0.98	-1.08	-0.88	100
4	-0.88	-0.97	-0.80	100

**Fig. 2:**
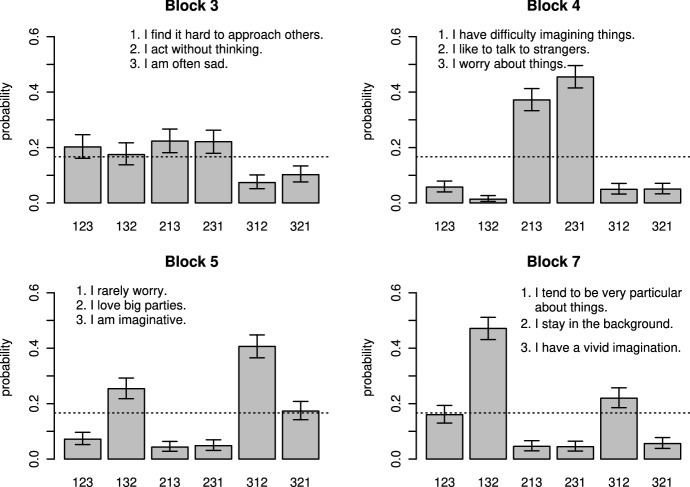
Probabilities for rank orders when faking in MFC-matched

The block fakabilities $\alpha _k$ show how well matching was achieved (i.e., whether it was clear which rank order to prefer when faking). As Table 4 shows, fakability was generally high, with 99 to 100% of participants predicted to fake for each block, but fakability still differed between the blocks. The rank-order probabilities provide additional information. Some exemplary rank-order probabilities are shown in Fig.  2. The other rank-order probabilities are shown in Figs. S17–S19. For all blocks, some rank orders were more desirable than others. Most blocks with intermediate fakability showed a pattern such as Block 3. Here, ranking the item "I am often sad" first was undesirable, whereas the remaining rank orders in which this item was ranked second or lowest were still desirable (Fig.  2). Thus, for such blocks, the number of possible rank orders when faking was limited to four instead of six. Among the highly fakable blocks, there were a few for which one rank order was more desirable than the other five, such as Block 7. Here, the order "I tend to be very particular about things," followed by "I have a vivid imagination," and finally "I stay in the background" was most desirable. For most of the highly fakable blocks, one item (out of three) was clearly preferred or not preferred, limiting the number of rank orders when faking to two. For example, in Block 4, it was desirable to rank the item "I like to talk to strangers" first, but it was not clear which of the other two items "I have difficulty imagining things" and "I worry about things" should be preferred. The opposite tendency appeared, for example, in Block 5. Here, it was desirable to rank the item "I love big parties" last. Thus, for some blocks, participants agreed on which item should be ranked first, whereas for other blocks, they agreed on which item should be ranked last.

#### Fakability of Mixed versus Matched Blocks

**Fig. 3:**
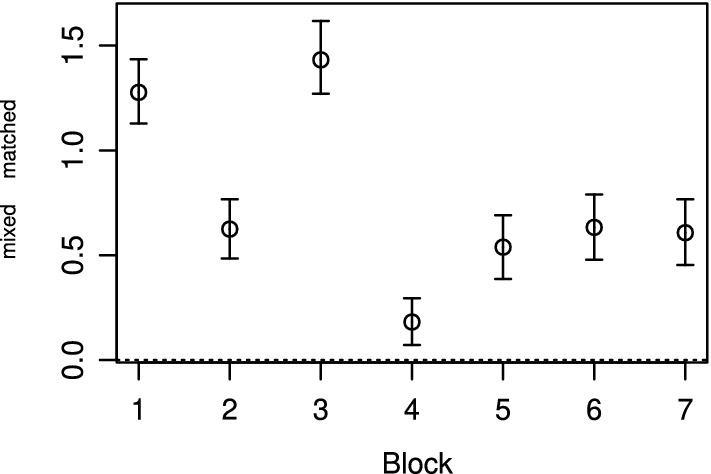
Differences in block fakability parameters $\alpha _k$ between MFC-mixed and MFC-matched

**Fig. 4:**
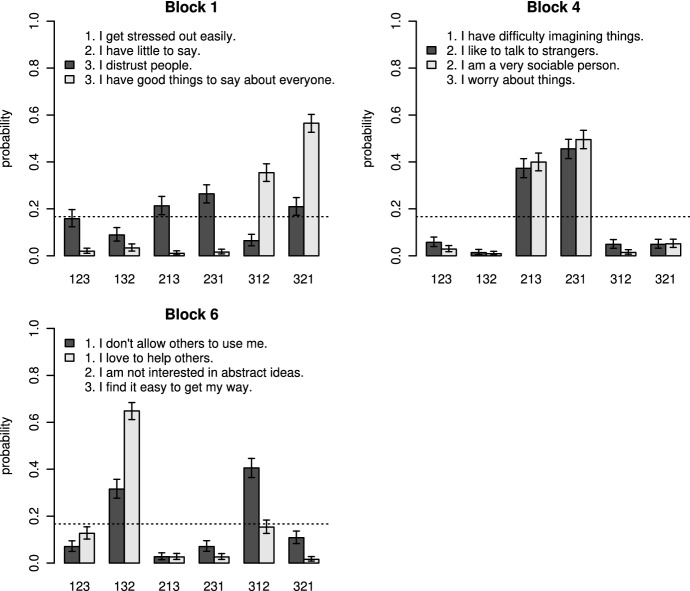
Probabilities for rank orders when faking in MFC-matched versus MFC-mixed

The block fakability parameters $\alpha _k$ were higher in MFC-mixed than in MFC-matched for all seven mixed blocks (Fig.  3). For Block 4, the block fakability parameters $\alpha _k$ were very high in both versions. The difference in block fakability parameters was descriptively higher for two out of the three socially undesirable Blocks 1 to 3, than for the neutral Blocks 4 to 7. Again, the rank-order probabilities derived from the rank-order parameters $\beta _{kr}$ provide additional information. For all blocks, including a highly socially desirable item resulted in some rank-order probabilities being close to zero. Figure 4 shows three exemplary patterns (for all blocks, see Fig.  S20). For some blocks, both rank orders favoring the highly desirable item were more likely in MFC-mixed than in MFC-matched. However, the pattern differed only slightly, when the highly desirable item in MFC-mixed replaced an item that was already desirable in MFC-matched, such as for Block 4 (Fig.  4). The pattern differed more markedly, when the highly desirable item in MFC-mixed replaced an undesirable item in MFC-matched, such as for Block 1. Here, in MFC-matched, the second item was most desirable and including a highly desirable third item in MFC-mixed led to overall high probabilities for the rank orders 3–1–2 and 3–2–1 (Fig. 4). For other blocks, only one of the rank orders favoring the highly desirable item was more likely in MFC-mixed. For example, for Block 6, the rank order 1–3–2 was more likely, but not the rank order 1–2–3.

### Discussion of Empirical Results

The sample had a strong tendency to fake as evidenced by a high faking trait mean. Such a sample is useful for evaluating maximum fakability during test construction. In real applicants or in clinical samples and in real instead of instructed faking situations, the tendency to fake will most likely be smaller (MacCann et al., 2011), resulting in fewer faked responses than in the current study.

The descriptive analysis of the block fakability parameters showed that matching did not work out for all blocks. Though items within blocks were carefully matched for desirability, some blocks were highly fakable. This might indicate that participants evaluate item desirability in a more fine-grained manner when items are combined into blocks rather than presented individually (see also Feldman & Corah, 1960). More generally, this is additional evidence of item interactions in MFC questionnaires (Lin & Brown, 2017), which make modeling on the block-level necessary. A second reason for high fakability might be that the faking scenario (applying for a place in a psychology master program) differed from the one used to assess item desirability (general social desirability).

Some blocks had intermediate fakability where two rank orders were clearly undesirable. According to the Faking Mixture model, which of the remaining rank orders to select when faking is random. However, several empirical phenomena could underlie the randomness captured by the model: Participants might have ranked the items randomly, according to differences in perceived desirability that were unsystematic across individuals, or according to their levels of the content traits. In the last case, blocks with intermediate fakability are informative about some of the measured traits. This could be investigated via the construct and criterion validity of traits estimated from blocks with intermediate fakability in a high-stakes situation. In an exploratory analysis, Wetzel et al. (2021) found decreases in criterion validity in a simulated high-stakes situation.

The current analysis showed that beyond the overall block fakability $\alpha _k$, the rank-order probabilities provide additional information. They show which items are more desirable and which comparisons might still remain informative about the content trait. This information could be used during questionnaire development to modify blocks by removing or replacing items or to discard whole blocks. For example, when the rank-order probabilities show that one item is clearly preferred over the others, this item could be removed from the block.

The comparison of MFC-mixed and MFC-matched showed two things: First, matching was worth the effort, because matched blocks were less fakable than mixed blocks in all seven cases. Second, matching based on the desirability of the individual items was not sufficient, because item interactions were observed on the block-level. It is therefore recommended to first match items for desirability and then to examine the fakability of the resulting MFC blocks.

In line with the trait shift approach to estimating the fakability of MFC questionnaires (Wetzel et al., 2021), the analysis with the Faking Mixture model showed that the MFC-matched version of the questionnaire was fakable to some extent and that the MFC-mixed version was more fakable. Applying the Faking Mixture model additionally showed which particular blocks were fakable and that it was truly the unmatched items that increased fakability in MFC-mixed.

## General Discussion

In this paper, I developed the Faking Mixture model, which is, to my knowledge, the first approach to modeling faking in the MFC format that allows the fakability of individual blocks to vary. A simulation study showed that the parameters of the Faking Mixture model could be recovered well under relevant conditions. Applying the Faking Mixture model to empirical data showed that matching based on the desirability ratings of the individual items was necessary but not sufficient to create an MFC questionnaire that can optimally reduce faking. Modeling fakability on the block-level allowed item context effects to be discovered within blocks. The Faking Mixture model can be used to reduce fakability during MFC test construction.

### Faking on the Block-Level

For practical applications, the trait shift approach and the Faking Mixture model complement each other, because the former focuses on the person level and the latter on the item level: Whereas the trait shift approach can show effects of faking on trait estimates, the Faking Mixture model can show which blocks are more or less fakable. Moreover, the Faking Mixture model complements methods of matching and assessing item desirability because it allows fakability to be estimated on the basis of responses to MFC blocks. The empirical validation showed that this approach is necessary because there were desirability differences that would not have been expected on the basis of the desirability ratings of the individual items. Using the results of the Faking Mixture model, test constructors can, for example, decide to keep only blocks with low fakability, or they can modify blocks by removing or replacing items that differ largely in their desirability from the others. Whereas the same can be achieved by tabulating frequencies of rank orders for honest versus faking conditions, the Faking Mixture model accounts for individual differences in the tendency to fake, which characterize most real assessment situations (Kuncel et al., 2012).

There are at least two differences between the response process for the rating scale versus the MFC format that make a mixture model on the block level parsimonious and necessary: First, because of the complexity of trait estimation, a shift model on the item level would be difficult if not impossible to identify. Second, a desirable rank-order cannot reasonably be determined without empirical data because item properties can change when items are combined into blocks (Lin & Brown, 2017).

### Limitations

One limitation of the Faking Mixture model is that the tendency to fake does not differ across content domains, as there is only one faking trait that is uncorrelated with all other traits. However, this limitation is probably not very critical because several authors have argued that when socially desirable responding or faking is present, scales that are otherwise multidimensional end up showing a one-factor structure (e.g., Guenole et al., 2018; MacCann et al., 2011; van der Linden et al., 2010). Moreover, differential desirability of the content traits can still be captured by the rank-order probabilities.

In the Faking Mixture model, as in the current models for the faking of rating scales, perceived item desirability is fixed across individuals. A model that allows perceived item desirability to vary across individuals would be difficult to identify, even if the model is theoretically plausible.

Usually the fakability of a questionnaire is investigated either by using instructed (induced) faking or by comparing two samples (e.g., applicants and incumbents). Instructed faking allows researchers to estimate maximum fakability, but it is not fully representative of real applicant situations, for example, because real applicants might have goals other than faking (Kuncel et al., 2012). Comparing applicants and incumbents allows researchers to estimate how much faking occurs in real settings, but there is no guarantee that the differences are due only to faking. To apply the Faking Mixture model, given the current hardware and software resources, response probabilities under a non-faking situation are needed. These are most easily obtained with instructed faking. However, there are a few studies that have used within-subjects data from naturally occurring contexts. Gordon and Stapleton (1956) compared the responses of high-school students who first filled out a questionnaire for guidance on a job search and some months later when they were actually seeking employment. Trent et al. (2020) compared the responses of army applicants with their later responses when they had been accepted into the army. Such a design would be optimal for the Faking Mixture model. Alternatively, a two-step procedure would be possible: First, instructed faking could be used to obtain estimates of item parameters and block fakabilities. Second, the faking trait and the content traits of another sample could be estimated with block and item parameters fixed to the estimates obtained in the first step. This is possible because, for the second step, only person parameters have to be estimated, which can be implemented in R. In this way, the Faking Mixture model could be used to obtain both the faking and the content trait estimates of real applicants.

The faking trait is incorporated in the Faking Mixture model to capture variance between individuals, but this variance is probably small in most cases, similar to response style traits (Böckenholt & Meiser, 2017). To assess the validity of the faking trait, a context in which it shows higher variance and therefore higher reliability is needed.

### Future Research Directions

There are several ways to assess item desirability in the literature: Some researchers use item intercepts, estimated under linear factor models or ideal-point models, or raw item means, from a previous administration of the item set as indicators of item desirability (e.g., Guenole et al., 2018). Alternatively, they compute the difference between item intercepts obtained under instructions to respond honestly versus to fake (e.g., Lee et al., 2018; Ng et al., 2020). Others take a more explicit approach and have an external group rate the desirability of each item, either in general (e.g., Wetzel & Frick, 2020) or for a specific scenario, or they combine the two approaches (e.g., Heggestad et al., 2006; Jackson et al., 2000). The Faking Mixture model could be used to investigate which type of desirability estimate and matching provide the smallest fakability. Further, it could be used to investigate the effect of item keying on fakability because this issue has been raised by several authors (Bürkner et al., 2019; Morillo et al., 2016).

Due to its implementation in a Bayesian framework, the Faking Mixture model is flexible for incorporating and testing additional assumptions. For example, in the empirical validation, differences in the fakability of matched versus unmatched blocks were quantified and tested. Future research could continue this avenue, for example, by testing whether fakability and rank-order probabilities are similar across different faking contexts.

The Faking Mixture model incorporates the assumption that respondents are more likely to respond honestly when the block is less fakable. However, it is an empirical question whether they actually respond honestly, or whether they retrieve further information about desirability.

In an exploratory analysis, Wetzel et al. (2021) compared the criterion validity of responses on rating scales and MFC blocks of matched and mixed desirability under instructed faking. To further validate the Faking Mixture model, future research could investigate whether less fakable blocks show higher criterion or construct validities.

To my knowledge, this is the first mixture model for MFC data with a mixture on the block level. For rating scales, mixture models were proposed not only for faking and social desirability (Böckenholt, 2014; Leng et al., 2019) but also for other response biases, such as acquiescence (Plieninger & Heck, 2018). Models with a discrete mixture (constant across items) have been used for response styles in general (for an overview, see Henninger & Meiser, 2020). Future research could develop models for other response biases, such as careless responding in MFC data with a mixture on the block-level.

Further, the Faking Mixture model could be populated with other IRT models such as the generalized graded unfolding model for rank data (Hontangas et al., 2015; Lee et al., 2019), or models for rating scale data. For rating scale data, because the response probabilities can be estimated in most common software programs, the model could even be applied to data from a high-stakes context only. However, in this design, the model might capture not only faking but also other preferences for categories that are also constant across the sample.

In this paper, I proposed the Faking Mixture model, an IRT model for faking on MFC questionnaires. The empirical model validation showed that to construct an MFC questionnaire that can optimally reduce faking, we need to both match items on desirability and examine the fakability of the resulting MFC blocks. The Faking Mixture model can be used for the latter and may thus become a valuable tool for MFC test construction. I hope that the Faking Mixture model can provide avenues for future discussion and research on item desirability, faking and the MFC format.

## Supplementary Information

Below is the link to the electronic supplementary material.

Supplementary material 1 (pdf 369 KB)
