# Supplementary material for: Modeling Faking in the Multidimensional Forced-Choice Format: The Faking Mixture Model
Source: Psychometrika. 2021 Dec 20;87(2):773–94. doi: 10.1007/s11336-021-09818-6 (PMC9166892; doi:10.1007/s11336-021-09818-6)
Supplement: Supplementary file 1 — Supplementary material 1 (pdf 369 KB) [file 11336_2021_9818_MOESM1_ESM.pdf]

Supplemental Online Material for

Modeling Faking in the Multidimensional Forced-Choice Format - The Faking Mixture Model

Supplemental Online Material for
Modeling Faking in the Multidimensional Forced-Choice Format - The Faking Mixture Model

Boxplots for the Dependent Variables in the Simulation Study by Condition

Main Parameters

Figure S1

Mean bias for the faking trait $\boldsymbol{\theta}$ *in the simulation study by condition*

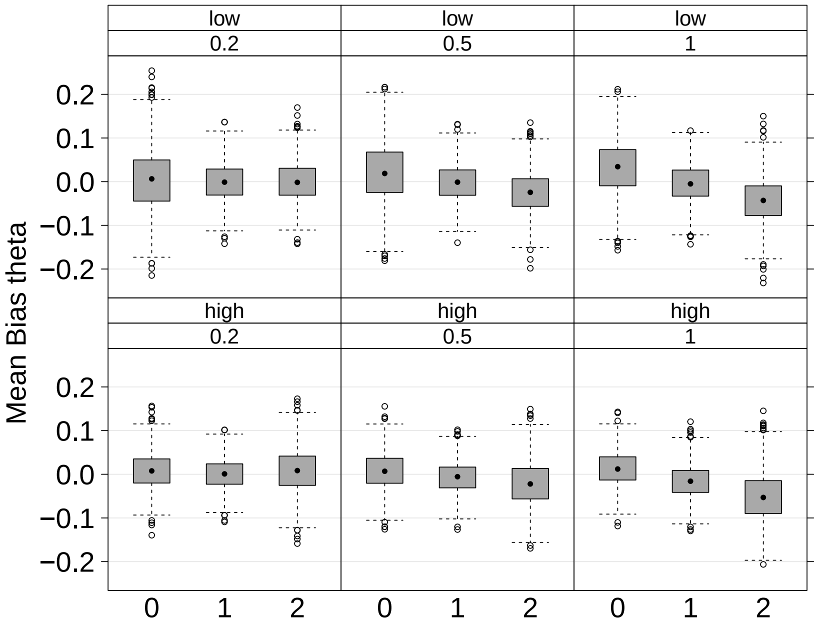

Note. low = low fakability, high = high fakability; 0.2, 0.5, 1.0 = faking trait variance

Figure S2

SD of bias for the faking trait $\boldsymbol{\theta}$ *in the simulation study by condition*

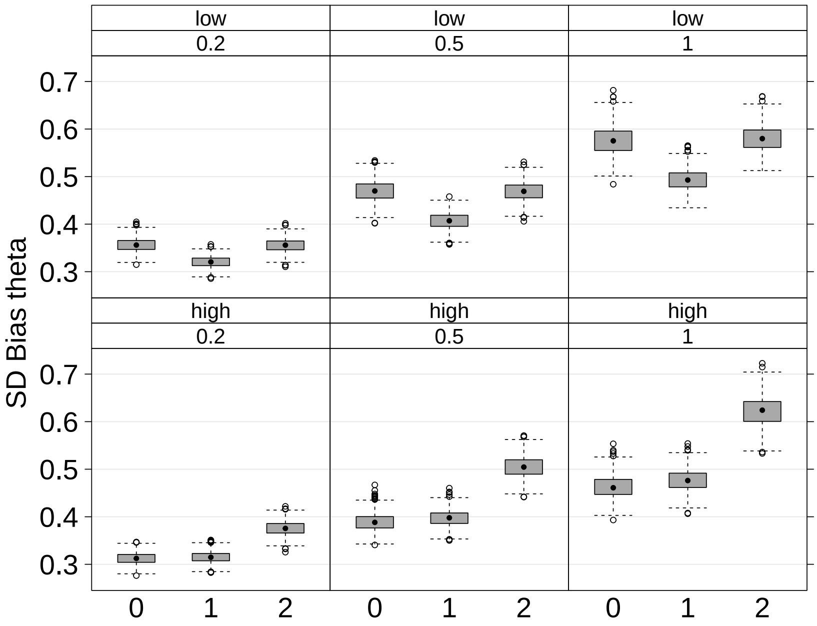

Note. low = low fakability, high = high fakability; 0.2, 0.5, 1.0 = faking trait variance

Figure S3

Coverage for the faking trait $\boldsymbol{\theta}$ *in the simulation study by condition*

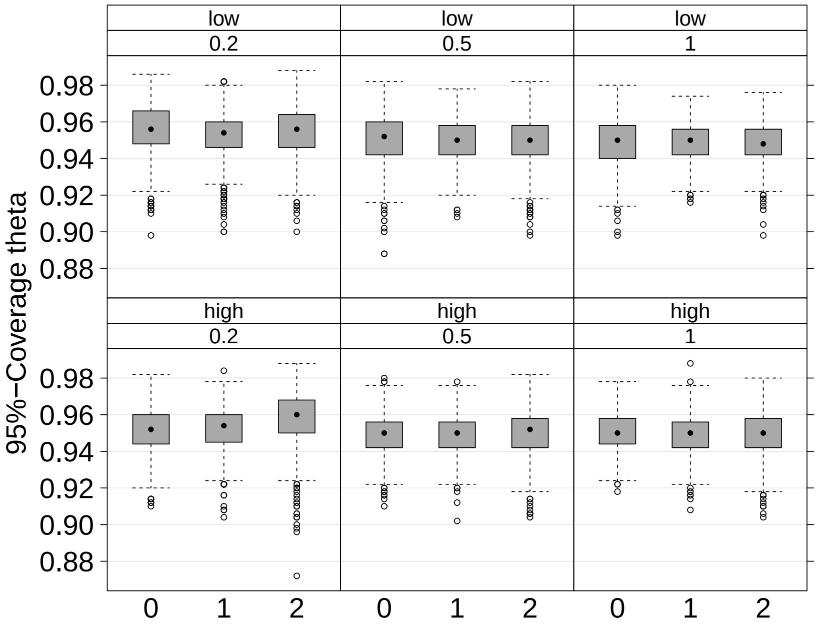

Note. low = low fakability, high = high fakability; 0.2, 0.5, 1.0 = faking trait variance

Figure S4

Mean bias for the rank-order parameters β_{kr} in the simulation study by condition

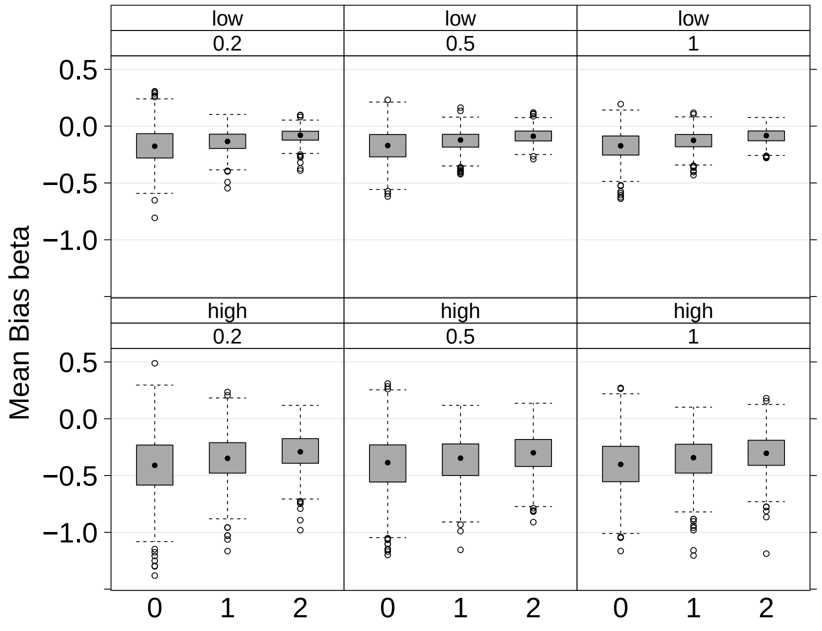

Note. low = low fakability, high = high fakability; 0.2, 0.5, 1.0 = faking trait variance

Figure S5

SD of bias for the rank-order parameters β_{kr} in the simulation study by condition

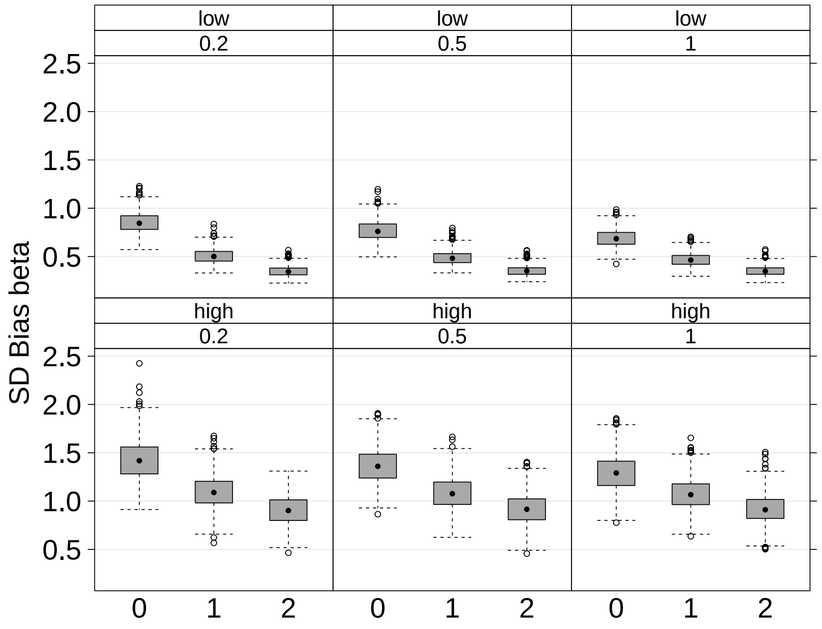

Note. low = low fakability, high = high fakability; 0.2, 0.5, 1.0 = faking trait variance

Figure S6

Coverage for the rank-order parameters β_{kr} in the simulation study by condition

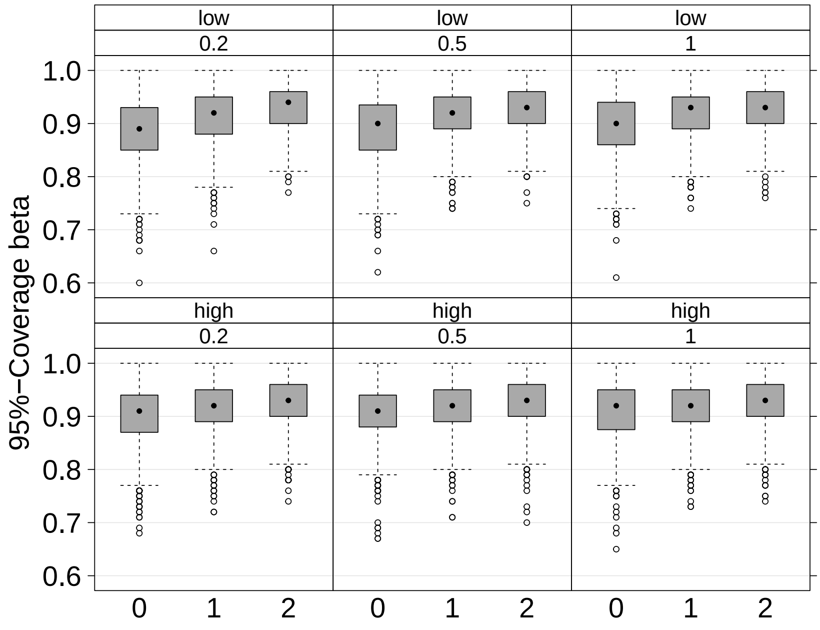

Note. low = low fakability, high = high fakability; 0.2, 0.5, 1.0 = faking trait variance

Hyperparameters

Figure S7

Bias for the faking trait mean $Mean(\boldsymbol{\theta})$ in the simulation study by condition

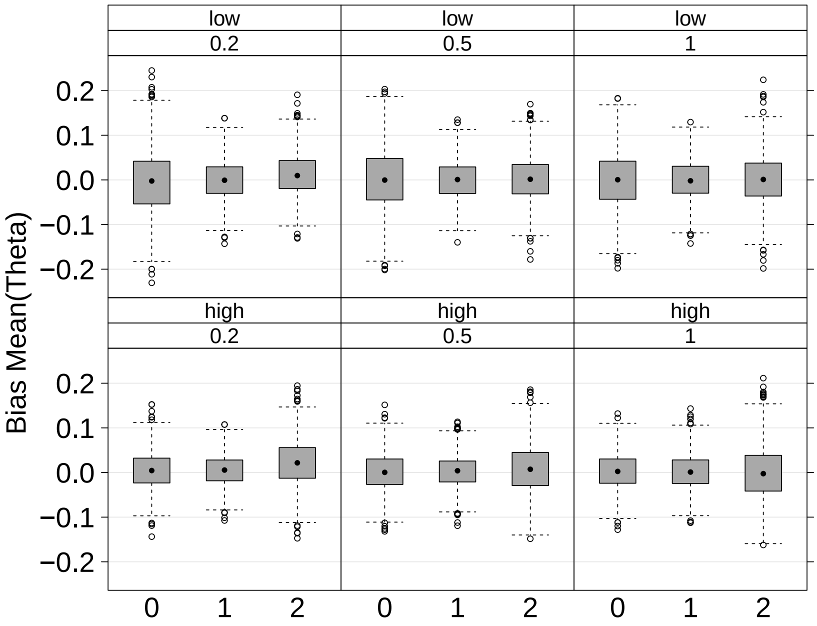

Note. low = low fakability, high = high fakability; 0.2, 0.5, 1.0 = faking trait variance

Figure S8

Bias for the faking trait variance $Var(\boldsymbol{\theta})$ in the simulation study by condition

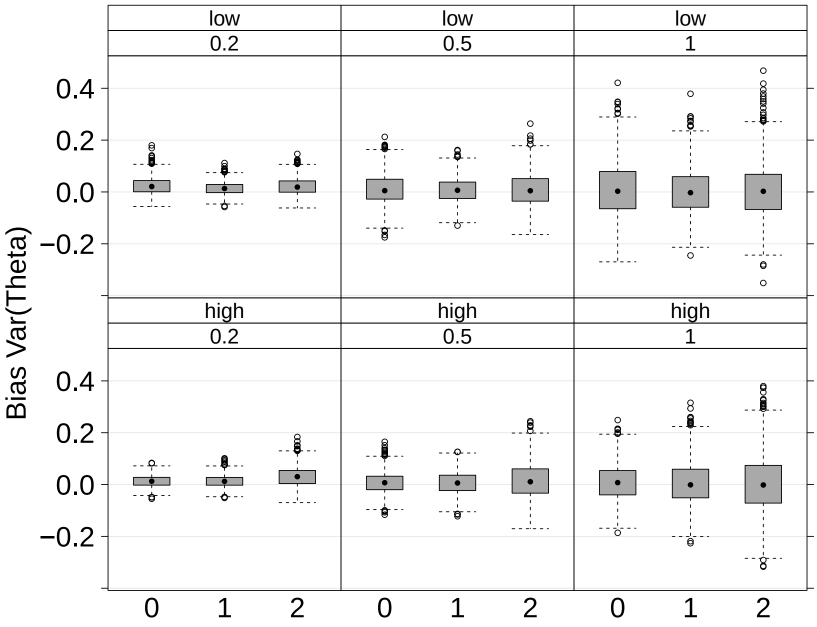

Note. low = low fakability, high = high fakability; 0.2, 0.5, 1.0 = faking trait variance

Figure S9

Bias for the variance of the rank-order parameters $Var(\boldsymbol{\beta})$ in the simulation study by condition

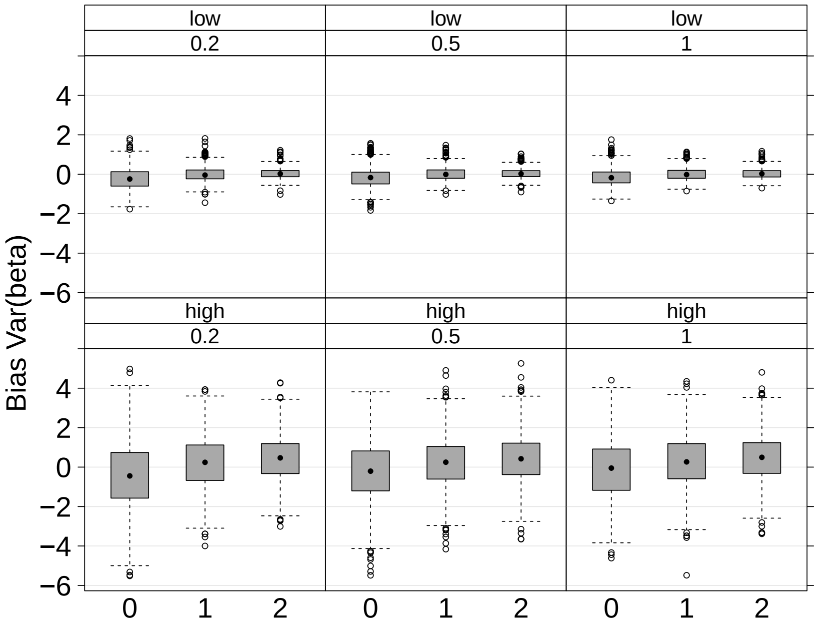

Note. low = low fakability, high = high fakability; 0.2, 0.5, 1.0 = faking trait variance

Derived Parameters

Figure S10

Mean bias for the block fakability parameters α_k in the simulation study by condition

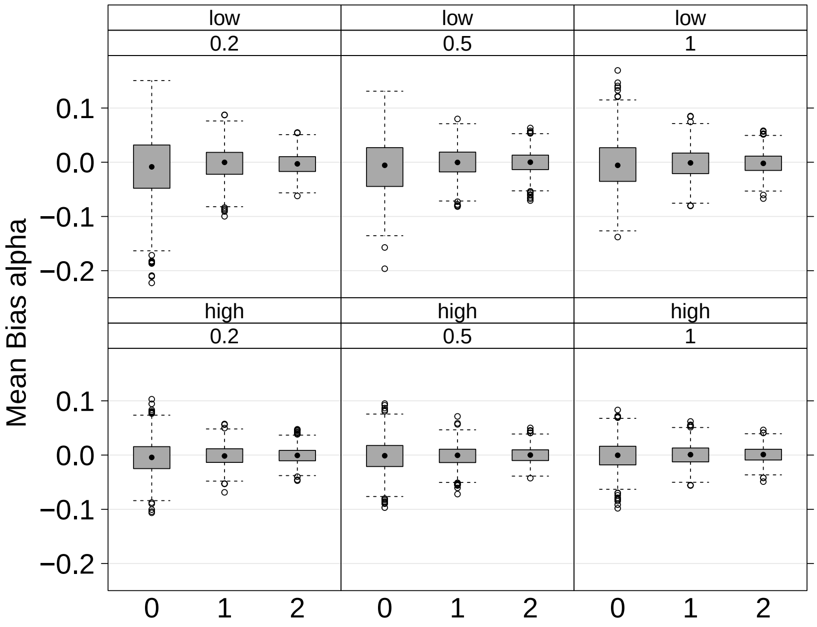

Note. low = low fakability, high = high fakability; 0.2, 0.5, 1.0 = faking trait variance

Figure S11

SD of bias for the block fakability parameters α_k in the simulation study by condition

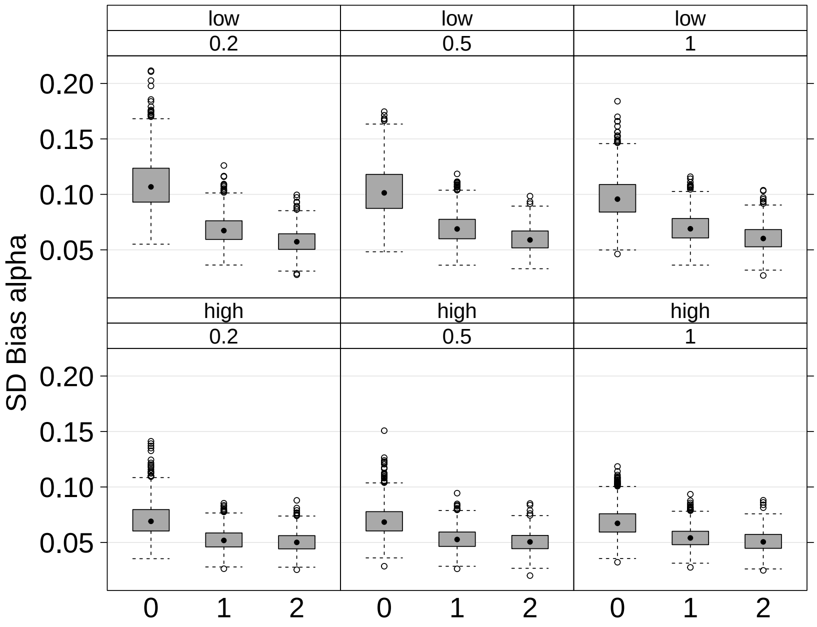

Note. low = low fakability, high = high fakability; 0.2, 0.5, 1.0 = faking trait variance

Figure S12

Coverage for the block fakability parameters α_k in the simulation study by condition

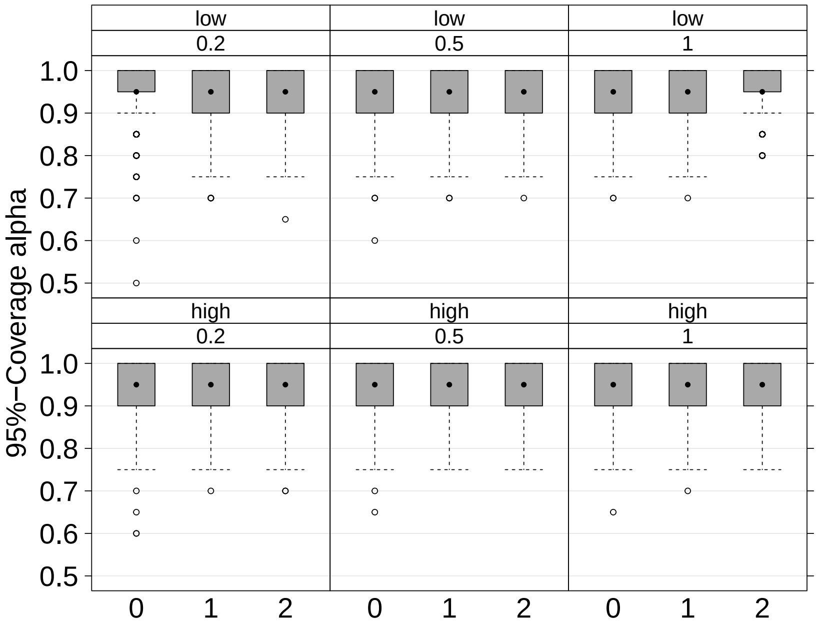

Note. low = low fakability, high = high fakability; 0.2, 0.5, 1.0 = faking trait variance

Figure S13

Correlations between true and estimated values for the rank-order probabilities $P(X_k = r|F_{jk} = 1)$ in the simulation study by condition

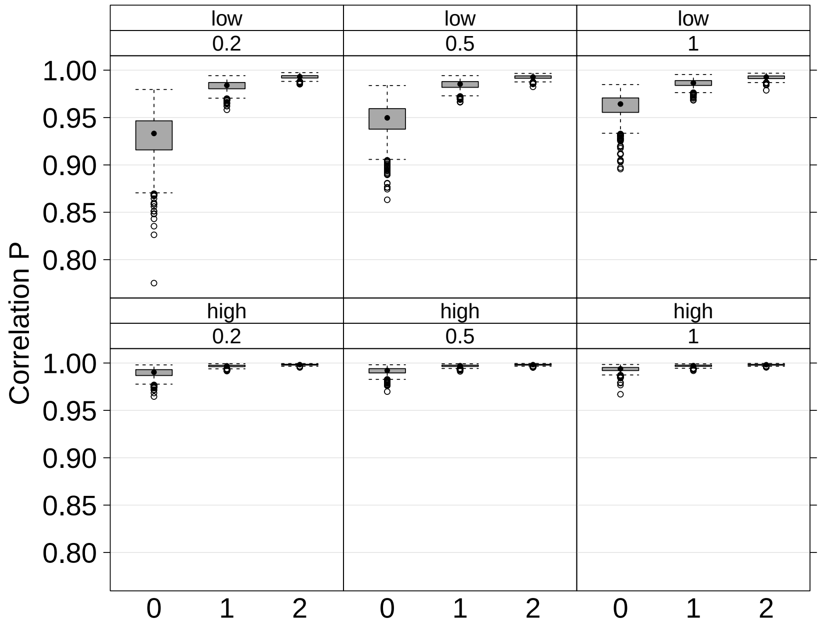

Note. low = low fakability, high = high fakability; 0.2, 0.5, 1.0 = faking trait variance

Figure S14

Mean bias for the rank-order probabilities $P(X_k = r|F_{jk} = 1)$ in the simulation study by condition

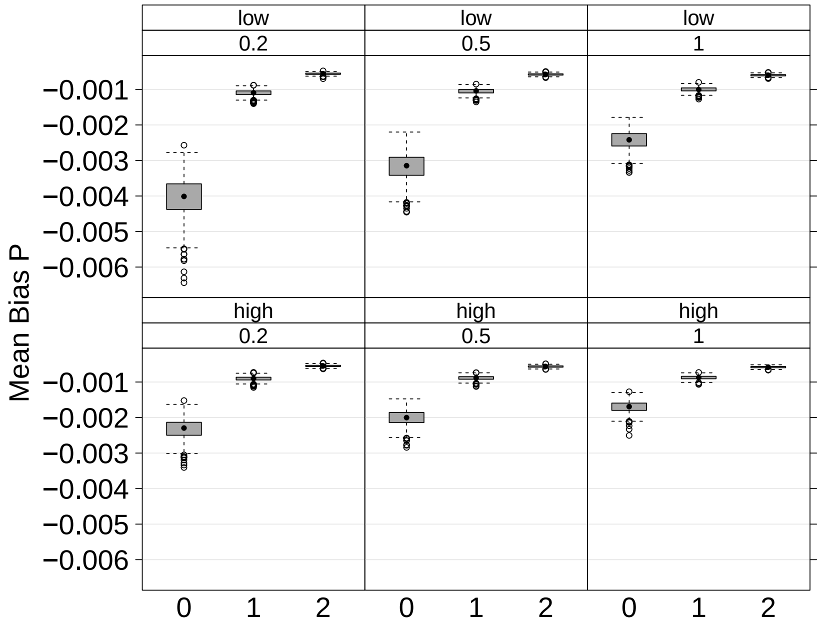

Note. low = low fakability, high = high fakability; 0.2, 0.5, 1.0 = faking trait variance

Figure S15

SD of bias for the rank-order probabilities $P(X_k = r|F_{jk} = 1)$ in the simulation study by condition

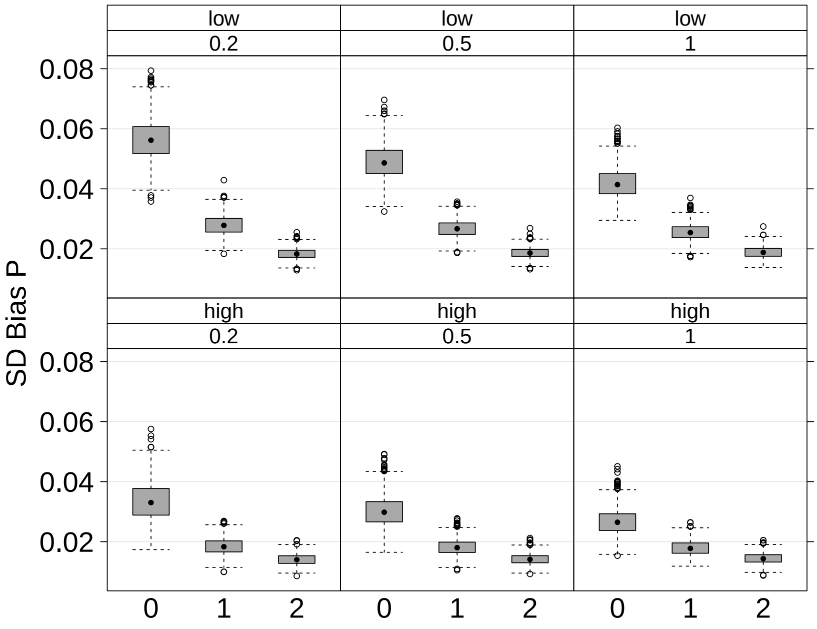

Note. low = low fakability, high = high fakability; 0.2, 0.5, 1.0 = faking trait variance

Figure S16

Coverage for the rank-order probabilities $P(X_k = r|F_{jk} = 1)$ in the simulation study by condition

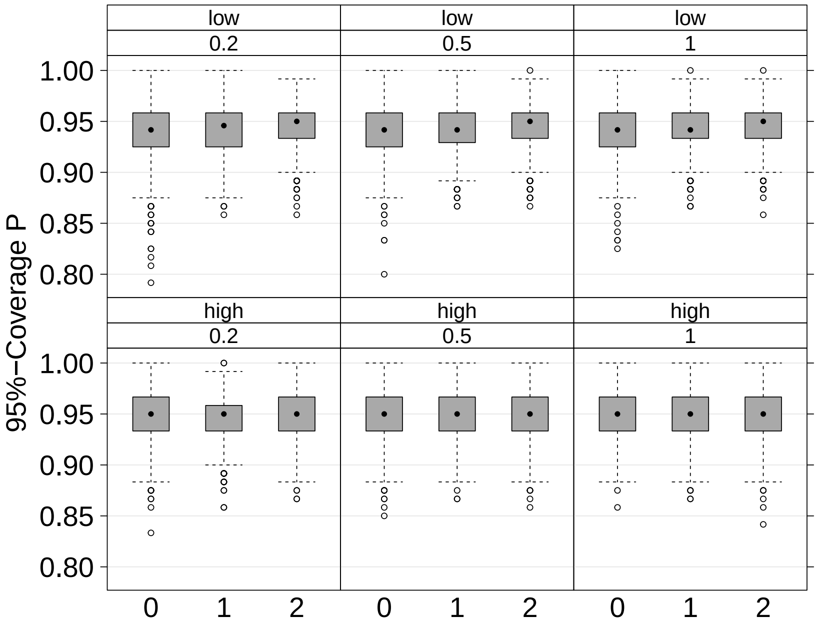

Note. low = low fakability, high = high fakability; 0.2, 0.5, 1.0 = faking trait variance

Figures for all Blocks in the Empirical Validation

Figure S17

Probabilities for rank orders when faking in MFC-matched for Blocks 1-8

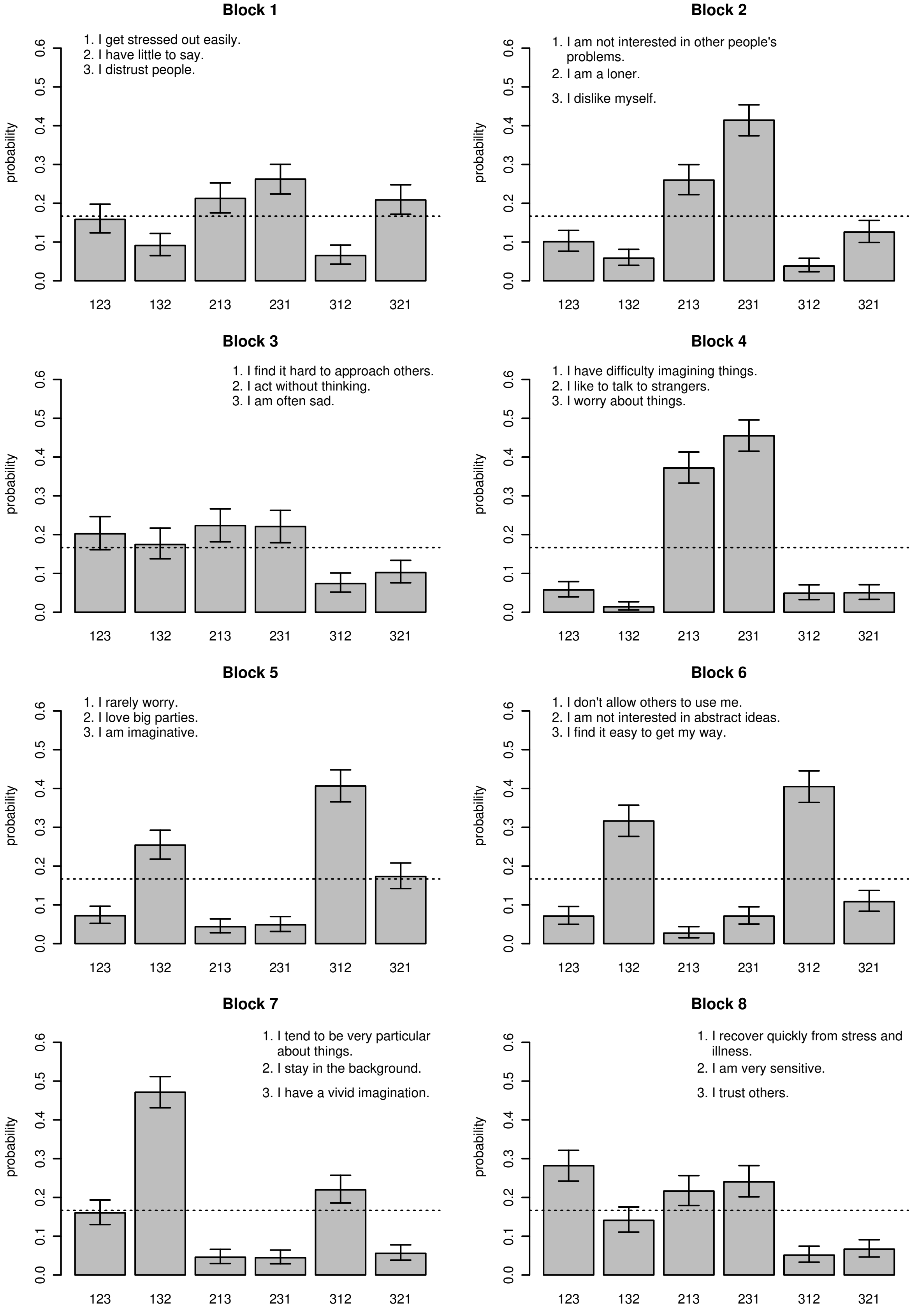

Note. The dotted line indicates where all rank orders are equally probable.

Figure S18

Probabilities for rank orders when faking in MFC-matched for Blocks 9-16

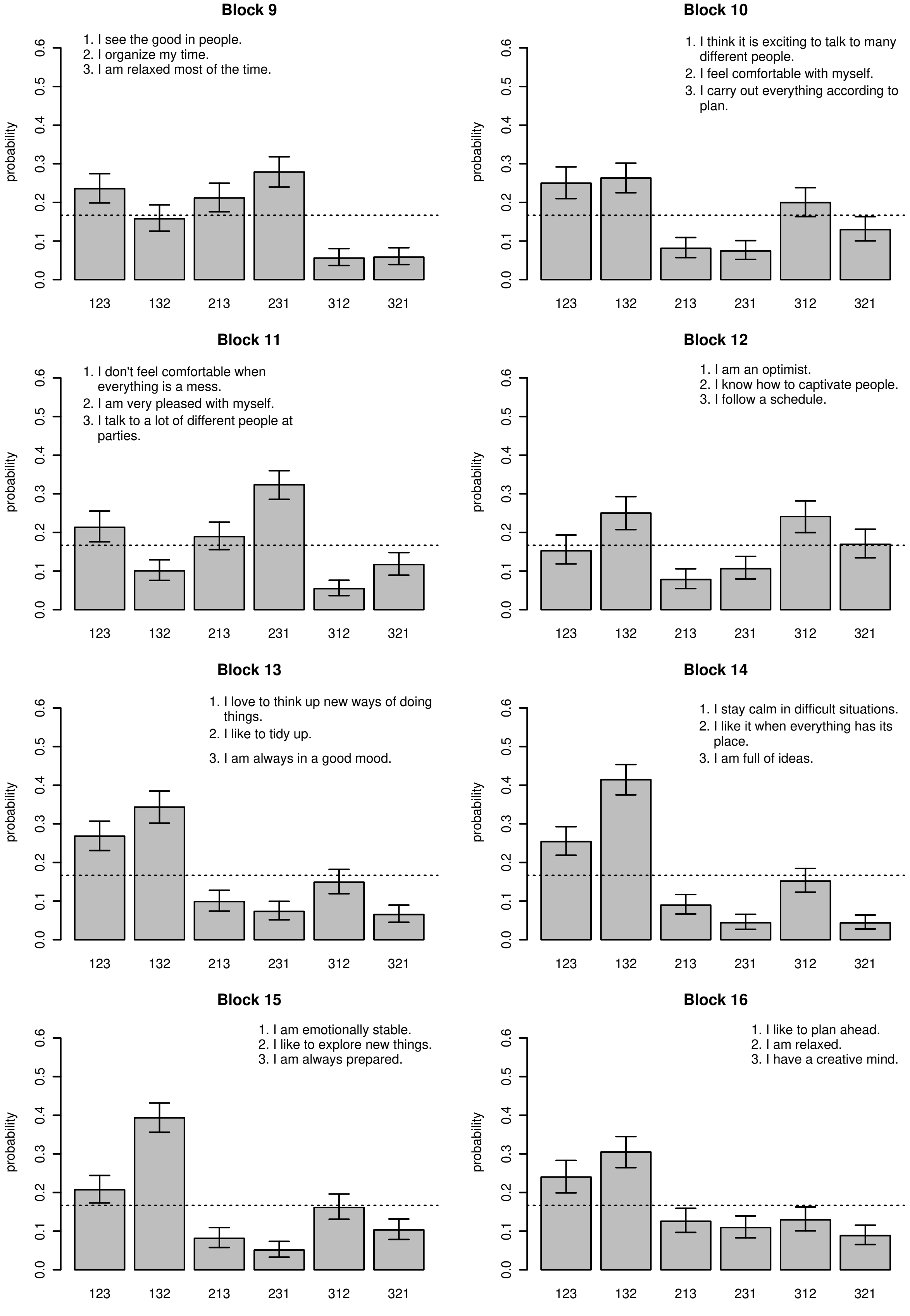

Note. The dotted line indicates where all rank orders are equally probable.

Figure S19

Probabilities for rank orders when faking in MFC-matched for Blocks 17-20

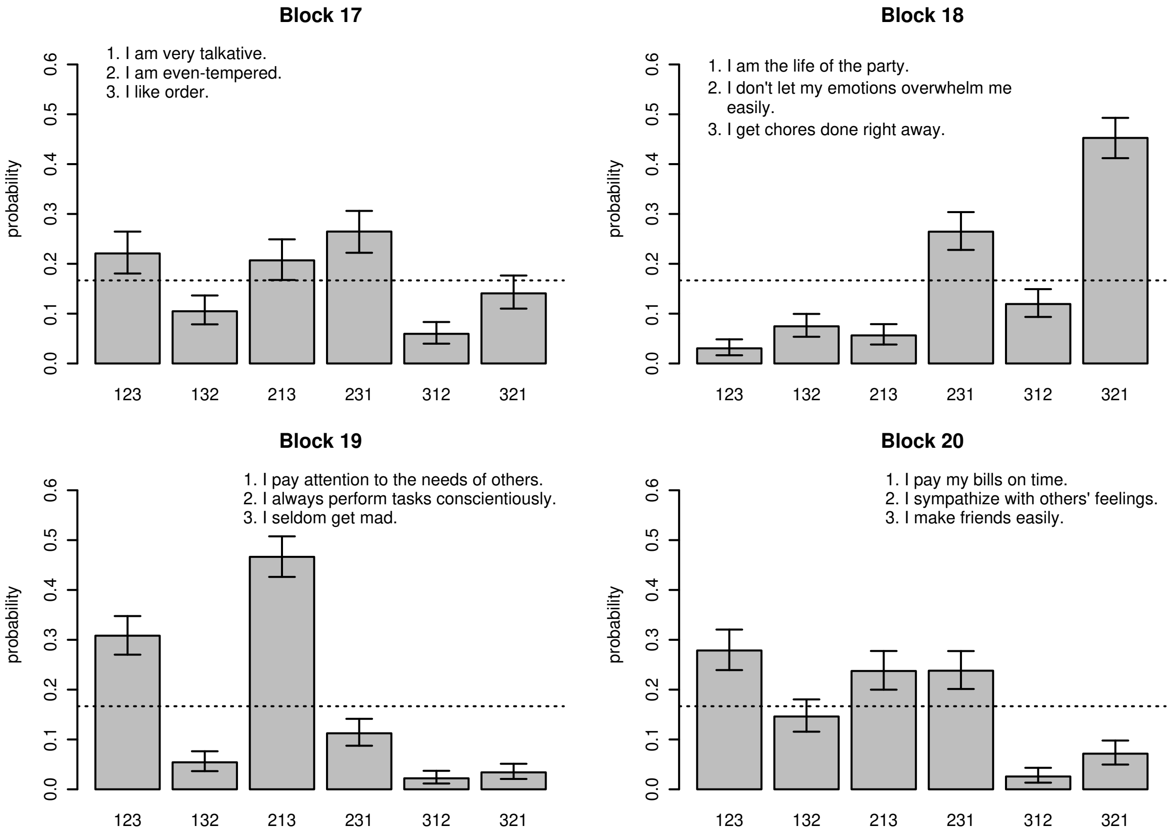

Note. The dotted line indicates where all rank orders are equally probable.

Figure S20

Probabilities for rank orders when faking in MFC-matched versus MFC-mixed

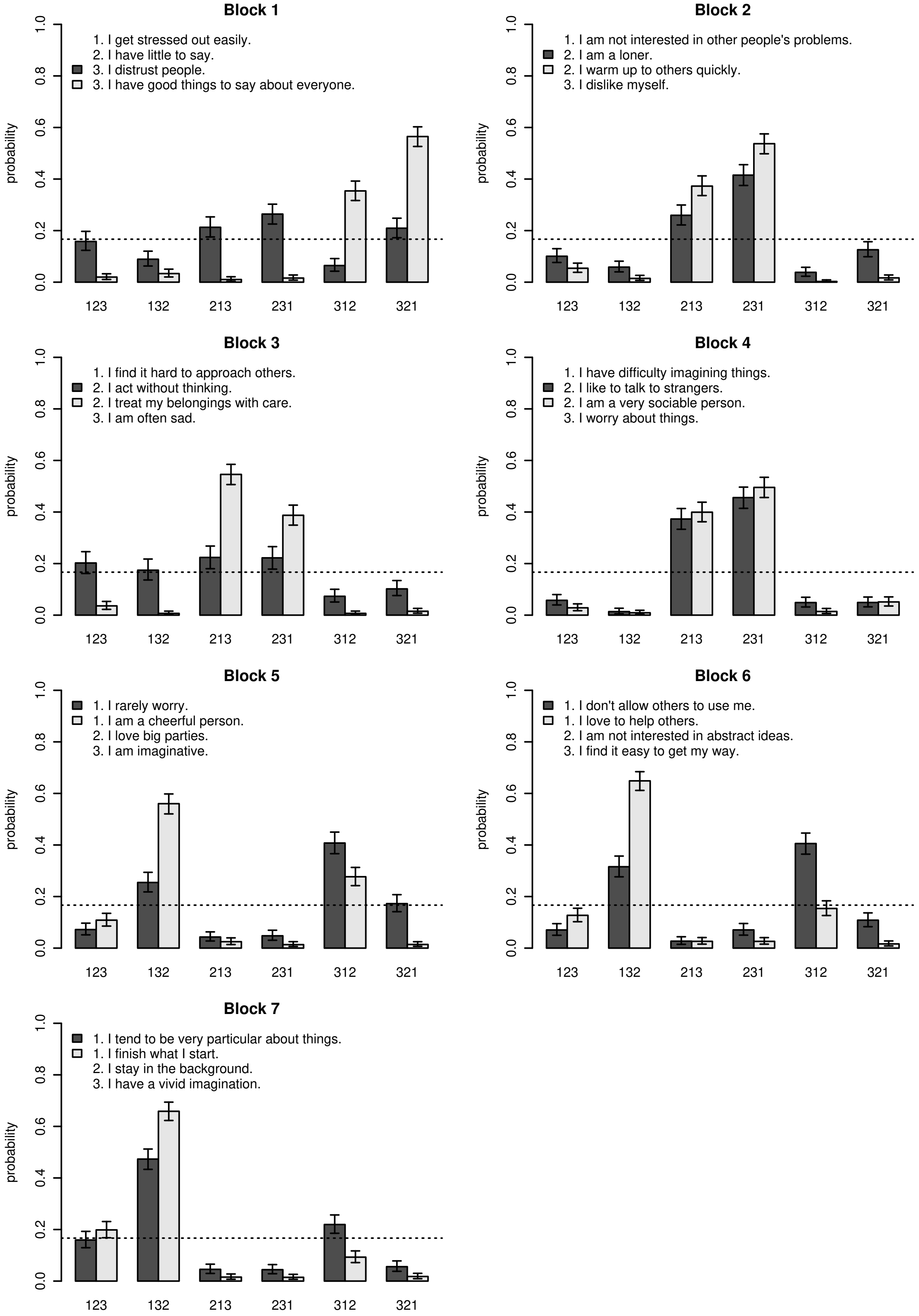

Note. The dotted line indicates where all rank orders are equally probable. Results for MFC-matched are depicted in dark-grey, for MFC-mixed in light-grey.
